# Endovascular versus open revascularization for acute arterial occlusive mesenteric ischemia: a retrospective single center analysis

**DOI:** 10.1007/s00423-025-03948-6

**Published:** 2025-12-11

**Authors:** Dominik Peter, Lars Kollmann, Annette Thurner, Amos Kroth, Ralph Kickuth, Christoph-Thomas Germer, Sven Flemming

**Affiliations:** 1 Department of General, Visceral, Transplantation, Vascular, and Pediatric Surgery, University Hospital Wuerzburg, Wuerzburg, Germany; 2 Department of Diagnostic and Interventional Radiology, University Hospital Wuerzburg, Wuerzburg, Germany; 3 Department of General, Visceral, Transplantation, Vascular and Pediatric Surgery, Center of Operative Medicine (ZOM), University Hospital of Wuerzburg, Wuerzburg, Germany

**Keywords:** Acute mesenteric ischemia, Mesenteric vascular occlusion, Bowel ischemia, Visceral artery revascularization, Vascular surgery, Endovascular therapy

## Abstract

**Purpose:**

Acute arterial occlusive mesenteric ischemia (AAOMI) is a life-threatening emergency associated with high mortality rates. Revascularization is a key component of multimodal therapy; however, the optimal initial treatment strategy, open surgical (OR) versus endovascular revascularization (ER), remains a subject of ongoing debate. This study aimed to compare outcomes between open and endovascular revascularization in patients with AAOMI.

**Methods:**

This retrospective single-center cohort study included all patients with AAOMI who underwent urgent revascularization between January 2004 and July 2024. Based on the initial revascularization method, patients were divided into two treatment groups: open surgical and endovascular. Outcomes included in-hospital mortality, bowel resection rate and extent, incidence of short bowel syndrome, and length of hospital and intensive care unit (ICU) stay.

**Results:**

Of the 100 patients included, 79 were initially treated with open revascularization and 21 with endovascular revascularization. In-hospital mortality was 48.1% (38/79) of OR patients and 33.3% (7/21) of ER patients (*p* = 0.227). 42 patients (53.2%) with open surgical treatment required bowel resection, compared to 10 patients (47.6%) with endovascular-first revascularization (*p* = 0.651). The median extent of bowel resection was 69 cm in the OR group and 71 cm in the ER group (*p* = 0.350). No differences could be detected regarding short bowel syndrome. Median hospital stay was 15 days in the open surgical cohort vs. 11 days in the endovascular cohort (*p* = 0.484). Median ICU stay was 5 days in the OR group and 4 days in the ER group (*p* = 0.172).

**Conclusion:**

Open surgical and endovascular revascularization resulted in comparable outcomes regarding in-hospital mortality, bowel resection, short bowel syndrome, and length of hospital and ICU stay in this retrospective cohort. Treatment decisions should be individualized based on occlusion type, patient condition, and institutional expertise. Prospective multicenter studies are warranted to further refine optimal management strategies for AAOMI.

## Introduction

Acute arterial occlusive mesenteric ischemia (AAOMI), characterized by an inadequate perfusion to the intestine, is a life-threatening and complex vascular emergency [1]. Significant visceral ischemia may lead to intestinal necrosis, septic complications with organ failure, and death [2]. The prognosis is known to be poor. Despite advances in diagnostics and therapy in recent years, mortality in acute mesenteric ischemia remains high, with rates exceeding 50% [3]. Non-specific symptoms resulting in delayed diagnosis and treatment, and commonly comorbid and elderly patients contribute to the fatal outcome [4].

The management of AAOMI is challenging. Early diagnosis and intervention are essential to improve outcomes in terms of mortality and the extent of bowel infarction and short bowel syndrome [5]. Revascularization of the superior mesenteric artery (SMA) represents the key component of a multimodal treatment approach [5]. Traditionally, AAOMI treatment consisted of open surgery. In past decades, endovascular interventions have been performed increasingly frequent [6, 7]. Some studies have shown that endovascular therapy is associated with decreased mortality and bowel resection rates compared to the open surgical approach [6–8].

Currently, there is no definite consensus on the optimal initial treatment modality for AAOMI, which is also based on the weak evidence. Thus, further studies in this challenging and multidisciplinary clinical field are strongly necessary to improve patient outcome in the future.

The objective of our study was to analyse clinical, radiological, and procedural data, and to compare the outcomes of open surgical revascularization (OR) versus endovascular revascularization (ER) as the first-line treatment strategy in patients with AAOMI.

## Materials and methods

### Study design and patients

This retrospective cohort study analyzed all patients diagnosed with AAOMI who underwent urgent revascularization at a single tertiary referral center between January 2004 and July 2024.

### Exclusion criteria

Patients were excluded if they had mesenteric venous thrombosis or non-occlusive mesenteric ischemia, received primary palliative care, or underwent bowel resection without an attempt at revascularization. Patients were also excluded if non-survivable bowel necrosis (NSBN) was identified during exploratory laparotomy, precluding curative treatment.

The decision to initiate palliative care was based on a combination of factors such as advanced age, severe comorbidities (e.g., dementia, poor performance status), prolonged disease course, unfavorable overall prognosis, and documented advance directives or explicit patient wishes.

The main reason for omitting revascularization was technical infeasibility, most commonly due to severe calcification of the mesenteric arteries and the absence of suitable distal target vessels for bypass grafting.

### Study groups

Based on the initial revascularization strategy, patients with AAOMI were divided into two treatment groups; open surgical revascularization and endovascular revascularization.

The open surgical group included patients with surgical or hybrid revascularization (ROMS, retrograde open mesenteric stenting) as first-line treatment, as both approaches involved initial laparotomy.

In the early phase of clinical practice, revascularization procedures were performed exclusively via open surgical techniques. With the development and progressive refinement of endovascular approaches, these techniques were gradually implemented in our center, with the first endovascular revascularization for AAOMI performed in 2012.

The choice of treatment strategy was determined by an interdisciplinary team comprising vascular surgeons, interventional radiologists, and visceral surgeons, based on clinical presentation, procedural feasibility, and institutional expertise.

### Treatment procedures

#### Endovascular revascularization

Endovascular mesenteric revascularization was performed under local or general anesthesia. Vascular access was typically obtained via the transfemoral route; in cases with anatomical or technical considerations, a transbrachial approach was used as an alternative. A 6 F to 8 F long guiding sheath (Destination Guiding Sheath, Terumo, Tokyo, Japan) was advanced to the level of the target vessels. Selective digital subtraction angiography (DSA) of the visceral arteries was performed to assess vascular anatomy and identify the site of occlusion. Lesion crossing was achieved using 0.014–0.035.014.035-inch guidewires (Choice PT Extra Support, Boston Scientific, Marlborough, MA, USA; Glidewire Advantage, Terumo, Tokyo, Japan; Radifocus, Terumo, Tokyo, Japan). Thrombembolic material was removed using rotational thrombectomy (Rotarex PRT device, BD, Franklin Lakes, NY, USA) and/or manual aspiration thrombectomy (BigLumen Aspiration catheter, Optimed, Ettlingen, Germany) techniques. The choice of technique was based on thrombus morphology, location, and procedural factors. Atherosclerotic stenoses were treated with percutaneous transluminal angioplasty (PTA) (Armada 14 and 18 PTA Catheter, Abbott Vascular, Redwood City, CA, USA) and stenting (0.014/0.018/0.035-inch) using different stents (Herculink, Abbott Vascular, Redwood City, CA, USA; Tsunami, Terumo, Tokyo, Japan; Palmaz Blue, Miami Lakes, FL, USA; BeSmooth, Bentley, Hechingen, Germany; BeGraft, Bentley, Hechingen, Germany). Hemostasis at vascular access sites was achieved either by manual compression or vascular closure devices.

#### Open surgical revascularization

Following laparotomy, the choice of revascularization technique was based on the underlying vascular pathology. Embolic occlusions were treated by balloon embolectomy using a 3- or 4-Fr Fogarty catheter. Thrombotic and atherosclerotic occlusions required adjunctive procedures. Depending on the location and extent of atherosclerotic disease, thrombendarterectomy (TEA) with patch angioplasty was performed for accessible lesions, ROMS was used for short-segment ostial stenoses, and mesenteric bypass was employed to manage long-segment occlusions.

The selection of bypass type (aorto-mesenteric, iliac-mesenteric, or spleno-mesenteric) was based on the donor vessel`s characteristics, ensuring adequate arterial inflow and technical feasibility for anastomosis.

Patch angioplasty graft materials included autologous great saphenous vein or xenogenic bovine pericardium (XenoSure Biologic Patch, LeMaitre Vascular, Burlington, MA, USA). Mesenteric bypass grafts were constructed using great saphenous vein, superficial femoral vein, or synthetic prostheses (Dacron: InterGard Vascular Graft, Intervascular SAS, Getinge Group, La Ciotat, France); ePTFE: Gore-Tex Vascular Graft, W. L. Gore & Associates, Flagstaff, AZ, USA). Vascular anastomoses were performed using Prolene 5.0 and 6.0 (Ethicon, Johnson & Johnson, Norderstedt, Germany) sutures.

### Data collection

Data were retrieved from the institutional medical records database. Collected variables included patient demographics, comorbidities, clinical presentation, diagnostics, treatment details, and clinical outcomes. Comparative analyses were conducted to assess differences between the two cohorts.

### Study endpoints

The primary endpoint was in-hospital mortality. Secondary endpoints included the bowel resection rate and extent, the development of short bowel syndrome, and the length of hospital and intensive care unit (ICU) stay.

### Ethical approval

Ethical approval was given by the local ethics committee (2025-392-dvhd).

### Statistical analysis

Statistical analyses were performed using IBM SPSS Statistics Version 29 (International Business Machines Corporation, Armonk, NY, USA). Descriptive data are presented as medians with range or interquartile range (IQR). Comparisons between groups were made using the Chi-square test, Fisher`s exact test, or Mann-Whitney U test, as appropriate. A two-sided p-value < 0.05 was considered statistically significant.

## Results

**Fig. 1:**
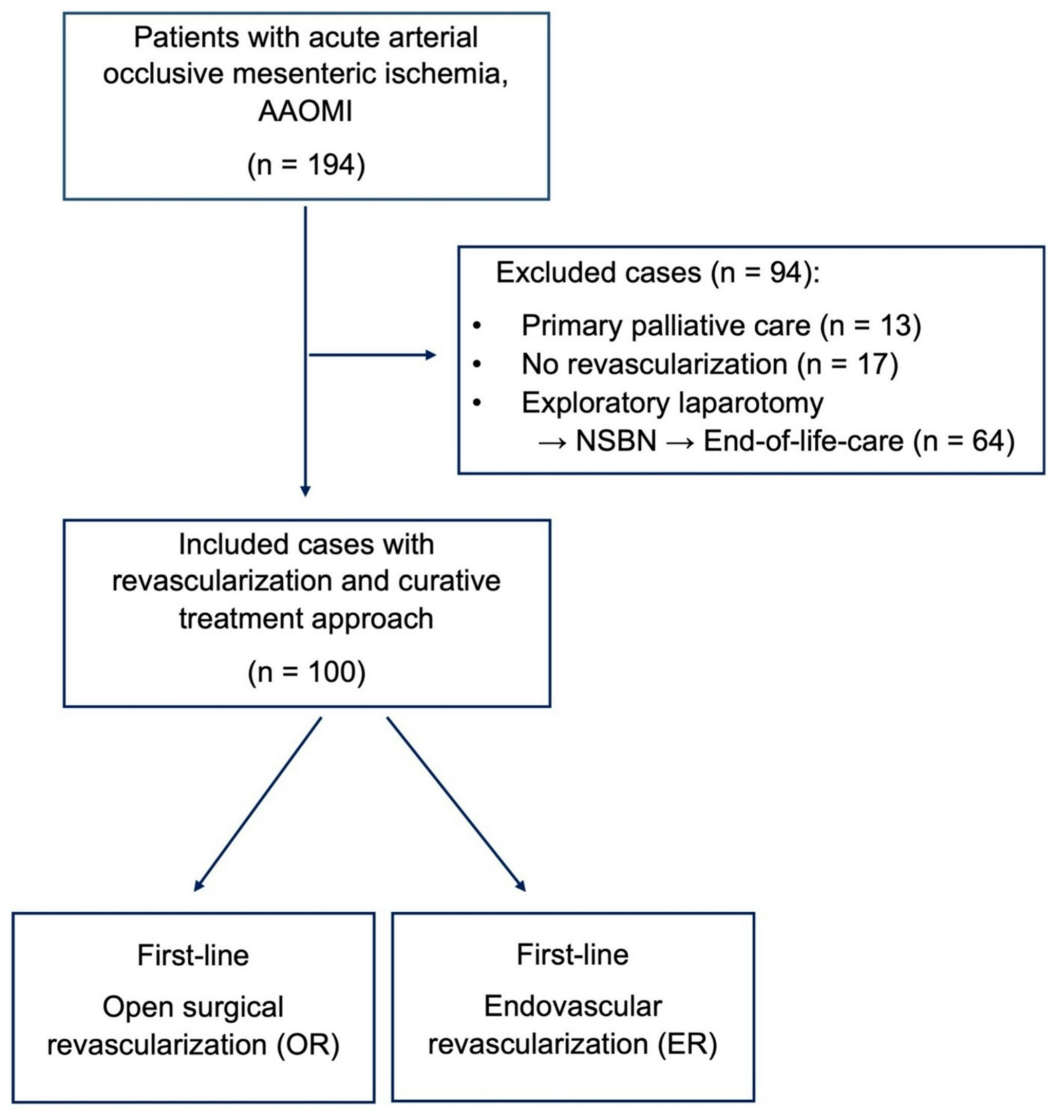
Study flowchart illustrating patient selection

A total of 194 patients with AAOMI were identified during the 20-year study period. Based on our inclusion and exclusion criteria, a total of 100 patients were eligible for final analysis. Overall, 79 patients (79%) initially underwent open revascularization, 21 patients (21%) were treated with endovascular revascularization (Fig. 1).

### Patient characteristics

The median age was 76 years (range 44–95) in the OR group and 70 years (range 52–92) in the ER group (*p* = 0.025). In the open revascularization group, 39.2% (31/79) were older than 80 years, compared to 19.1% (4/21) in the endovascular group (*p* = 0.122). 54.4% of the patients with OR and 61.9% of the patients with ER were female (*p* = 0.540).

The median Charlson Comorbidity Score (CCS) was 5.5 (range 1–12) in the total cohort, with a median of 6.0 (range 1–12) in the OR group and 5.0 (range 2–11) in the ER group (*p* = 0.114).

Previous visceral artery interventions due to chronic mesenteric ischemia (abdominal angina) were more frequent in the endovascular group (OR 5.1% vs. ER 33.3%; *p* = 0.001). Patients with ER also had a higher rate of previous bowel resections (OR 3.9% vs. ER 19.0%; *p* = 0.036).

Detailed patient characteristics, including cardiovascular risk factors, comorbidities, and anticoagulation status, are shown in Table 1.

**Table 1:** Patient characteristics

	Total *n* = 100 (100%)	Open *n* = 79 (79%)	Endovascular *n* = 21 (21%)	*p* value
Baseline				
Age, years, median (range)	75 (44–95)	76 (44–95)	70 (52–92)	**0.025**
Age ≥ 80 years	35 (35.0%)	31 (39.2%)	4 (19.1%)	0.122
Sex - Male - Female	44 (44.0%) 56 (56.0%)	36 (45.6%) 43 (54.4%)	8 (38.1%) 13 (61.9%)	0.540
Body mass index, kg/m², median (IQR)	26.7 (23.4–29.4)	27.0 (23.8–29.7)	24.7 (22.8–27.6)	0.211
Cardiovascular risk factors				
Hypertension No data	91 (92.0%)	73 (93.6%) 1	18 (85.7%)	0.240
Diabetes mellitus - insulin dependent No data	24 (24.5%) 10 (10.2%)	21 (27.3%) 10 (13.0%) 2	3 (14.3%) 0 (0.0%)	0.266 0.113
Dyslipidemia No data	54 (55.1%)	43 (55.8%) 2	11 (52.4%)	0.777
Smoking (current/any history) No data	38 (53.5%) 29	27 (50.9%) 26	11 (61.1%) 3	0.455
Comorbidities				
Atrial fibrillation No data	50 (50.5%)	42 (53.8%) 1	8 (38.1%)	0.200
Coronary artery disease No data	27 (27.3%)	20 (25.6%) 1	7 (33.3%)	0.482
Prior myocardial infarction No data	11 (11.2%)	8 (10.4%) 2	3 (14.3%)	0.698
Peripheral vascular disease, ≥ Fontaine stage II No data	28 (28.3%)	22 (28.2%) 1	6 (28.6%)	0.974
Chronic kidney disease (CKD), ≥ KDIGO stage III - dialysis (CKD V)	36 (36.0%) 4 (4.0%)	29 (36.7) 3 (3.8%)	7 (33.3%) 1 (4.8%)	0.775 1.000
Chronic obstructive pulmonary disease No data	28 (28.6%)	27 (35.1%) 2	1 (4.8%)	**0.006**
Cerebrovascular disease * No data	28 (28.6%)	22 (28.6%) 2	6 (28.6%)	1.000
History of Malignancy No data	26 (26.5%)	21 (27.3%) 2	5 (23.8%)	0.750
Dementia No data	5 (5.1%)	5 (6.5%) 2	0 (0.0%)	0.582
Charlson comorbidity score, median (range)	5.5 (1–12)	6.0 (1–12)	5.0 (2–11)	0.114
ASA classification ≥ III No data	64 (91.4%) 30	49 (94.3%) 27	15 (83.3%) 3	0.155
Medications				
Antiplatelet agents No data	54 (56.3%) 4	45 (59.2%) 3	9 (45.0%) 1	0.254
Anticoagulation agents No data	19 (19.8%) 4	15 (19.7%) 3	4 (20.0%) 1	1.000
Previous surgeries				
Previous bowel resection No data	7 (7.1%)	3 (3.9%) 2	4 (19.0%)	**0.036**
Previous visceral artery intervention/surgery	11 (11.0%)	4 (5.1%)	7 (33.3%)	**0.001**

Data are presented as n (%), median (range), or median (IQR)

* history of stroke or transient ischaemic attack, stenosis internal carotid artery ≥ 60%, previous carotid angioplasty and/or stenting

### Clinical presentation

In total, 58% of patients were secondary referrals from other hospitals. The median transfer distance was 33 km, ranging from 1.4 km to 86 km.

Most patients (68%) were admitted on working days; however, 65% were admitted beyond regular working hours. Treatment was performed outside of day shift hours in 70.9% of OR cases and 47.6% of ER cases (*p* = 0.045).

Delayed treatment (> 24 h from symptom onset) occurred in 48.4% of OR and 66.7% of ER cases (*p* = 0.172).

Abdominal pain was the leading clinical symptom, present in 97.8% overall. Diarrhea was reported in 27.8% of OR cases and 31.6% of ER cases (*p* = 0.744). Nausea and/or vomiting was more common in the ER group (78.9%) compared to OR (48.6%) (*p* = 0.018). Hematochezia occurred in 13.9% of OR and 36.8% of ER patients (*p* = 0.022).

Clinical signs of peritoneal irritation were observed in 51.4% of patients in the OR group and in 26.3% of patients in the ER group (*p* = 0.051).

Critical illness, defined as hemodynamic instability and/or catecholamine use, septic shock, organ failure, cardiopulmonary resuscitation, mechanical ventilation, or ICU treatment, was observed in a minority of patients (14%) with 15.2% in the OR group vs. 9.5% in the ER group (*p* = 0.728) (Table 2).

**Table 2:** Clinical presentation

	Total *n* = 100 (100%)	Open *n* = 79 (79%)	Endovascular *n* = 21 (21%)	*p* value
Logistical data				
Referral from another hospital	58 (58.0%)	46 (58.2%)	12 (57.1%)	0.929
Consultation - working day - weekend/public holiday	68 (68.0%) 32 (32.0%)	53 (67.1%) 26 (32.9%)	15 (71.4%) 6 (28.6%)	0.705
Out-of-office hours, admission	65 (65.0%)	52 (65.8%)	13 (61.9%)	0.738
Out-of-office hours, therapy	66 (66.0%)	56 (70.9%)	10 (47.6%)	**0.045**
Prior surgery/intervention < 30 days	22 (22.0%)	17 (21.5%)	5 (23.8%)	0.822
Time management				
Symptom onset to treatment - time interval < 24 h - time interval > 24 h No data	38 (47.5%) 42 (52.5%) 20	32 (51.6%) 30 (48.4%) 17	6 (33.3%) 12 (66.7%) 3	0.172
Clinical symptoms				
Abdominal pain No data	89 (97.8%) 9	70 (97.2%) 7	19 (100%) 2	0.463
Nausea/Vomiting No data	50 (54.9%) 9	35 (48.6%) 7	15 (78.9%) 2	**0.018**
Diarrhea No data	26 (28.6%) 9	20 (27.8%) 7	6 (31.6%) 2	0.744
Bloody diarrhea/Hematochezia No data	17 (18.7%) 9	10 (13.9%) 7	7 (36.8%) 2	**0.022**
Clinical examination				
Signs of peritoneal irritation No data	41 (46.1%) 11	36 (51.4%) 9	5 (26.3%) 2	0.051
Clinical status, preoperative				
Critical illness *	14 (14.0%)	12 (15.2%)	2 (9.5%)	0.728
- Intensive care unit stay, pre-AMI	11 (11.0%)	9 (11.4%)	2 (9.5%)	1.000
- Hemodynamic instability and/or catecholamine use	11 (11.0%)	10 (12.7%)	1 (4.8%)	0.450
- Mechanical ventilation	6 (6.0%)	5 (6.3%)	1 (4.8%)	1.000
- Resuscitation	2 (2.0%)	2 (2.6%)	0 (0.0%)	1.000

Data are presented as n (%)

* Hemodynamic instability and/or catecholamine use, septic shock/organ failure, resuscitation, mechanical ventilation, pre-ICU

### Laboratory and imaging evaluation

White blood cells, C-reactive protein (CRP), and lactate levels were elevated in both groups, with no significant differences between them. Overall, the median leukocyte count was 18,700/µL, the median CRP level was 10.3 mg/dL, and the median lactate level was 3.0 mmol/L.

In 94% of all cases, the diagnosis was made using computed tomography (CT) angiography.

Various causes of AAOMI were identified in the study population, with both embolic and non-embolic etiologies observed. An embolic origin was more frequently detected in the open surgery group (59.5%) compared to the endovascular group (38.1%), although this difference did not reach statistical significance (*p* = 0.080).

In the ER group, most lesions were located at the ostium (38.1%) or represented in-stent thromboses (28.6%). In-stent thrombosis occurred significantly more often in the ER group compared to the OR group (28.6% vs. 1.3%; *p* = 0.0003). In contrast, the OR group predominantly exhibited long-segment occlusions involving side branches (46.5% vs. 19%; *p* = 0.041).

**Fig. 2:**
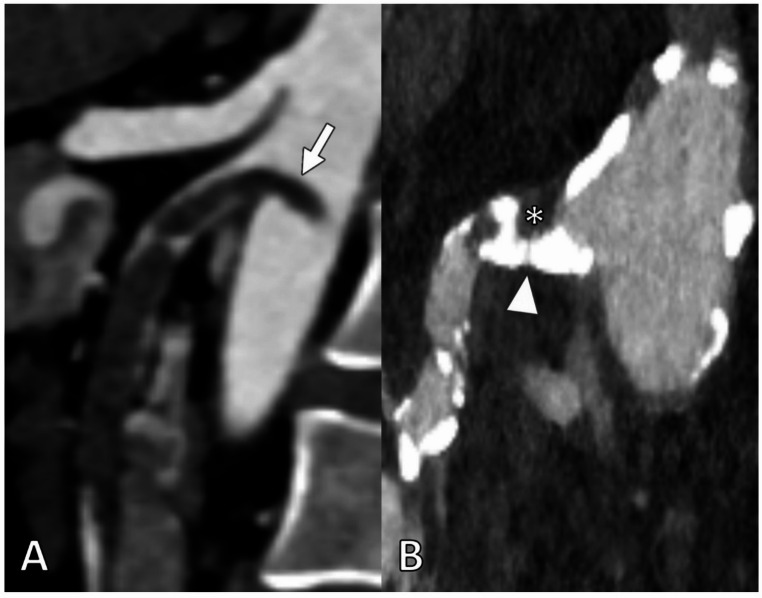
CT angiography in parasagittal views revealed two distinct pathological entities of AAOMI. (**A**) shows an embolic occlusion of the superior mesenteric artery, with the embolus protruding into the aortic lumen (arrow). Note the absence of atherosclerotic lesions. In contrast, (**B**) demonstrates a highly calcified atherosclerotic stenosis (arrowhead) at the SMA ostium, complicated by superimposed thrombotic occlusion (asterisk) due to plaque rupture

Further details on vascular status and occlusion characteristics are provided in Table 3. Exemplary CT scans of embolic and thrombotic entities of AAOMI are shown in Fig. 2.

**Table 3:** Laboratory and imaging evaluation

	Total *n* = 100 (100%)	Open *n* = 79 (79%)	Endovascular *n* = 21 (21%)	*p* value
Laboratory values				
White blood count, thousands/µL, median (IQR)	18.7 (13.2–23.6)	17.6 (12.5–23.6)	19.8 (17.1–23.6)	0.162
C-reactive protein, mg/dL, median (IQR)	10.3 (2.5–23.3)	9.4 (2.0–23.8.0.8)	15.0 (5.0–22.2.0.2)	0.394
Lactate level, mmol/L, median (IQR)	3.0 (2.0–4.7.0.7)	3.2 (2.0–4.7.0.7)	2.4 (1.9–4.8)	0.504
pH, median (IQR)	7.33 (7.26–7.39)	7.33 (7.26–7.39)	7.35 (7.24–7.40)	0.790
Lactate dehydrogenase, U/L, median (IQR)	307 (261–417)	303 (271–416)	312 (223–417)	0.637
Radiological findings				
Contrast-enhanced computed tomography	94 (94.0%)	73 (92.4%)	21 (100%)	0.193
Vascular status				
Probable entity, embolic cause	55 (55.0%)	47 (59.5%)	8 (38.1%)	0.080
Probable entity, subtypes				
- Embolic	55 (55.0%)	47 (59.5%)	8 (38.1%)	0.080
- Thrombotic	17 (17.0%)	14 (17.7%)	3 (14.3%)	1.000
- Arteriosclerotic	14 (14.0%)	10 (12.7%)	4 (19.0%)	0.485
- In-Stent-thrombosis	7 (7.0%)	1 (1.3%)	6 (28.6%)	**0.0003**
- Bypass occlusion	1 (1.0%)	1 (1.3%)	0 (0.0%)	1.000
- Isolated SMA dissection	2 (2.0%)	2 (2.5%)	0 (0.0%)	1.000
- Vasculitis (giant cell arteritis)	1 (1.0%)	1 (1.3%)	0 (0.0%)	1.000
- Unknown entity	3 (3.0%)	3 (3.8%)	0 (0.0%)	1.000
Occlusion level, SMA				
- Ostial	38 (41.3%)	30 (42.3%)	8 (38.1%)	0.734
- Proximal to the middle colic artery	8 (8.7%)	6 (8.5%)	2 (9.5%)	1.000
- Distal to the middle colic artery	1 (1.1%)	0 (0.0%)	1 (4.8%)	0.228
- Involving side branches	37 (40.2%)	33 (46.5%)	4 (19.0%)	**0.041**
- In-Stent/bypass occlusion	8 (8.7%)	2 (2.8%)	6 (28.6%)	**0.002**
No data		8		
Occlusion length, SMA, mm, median (IQR)	35 (23–45)	35 (23–44)	34 (23–45)	0.762
Visceral artery disease (NASCET)				
- None	38 (41.3%)	31 (43.7%)	7 (33.3%)	0.398
- Single	16 (17.4%)	13 (18.3%)	3 (14.3%)	1.000
- Double	13 (14.1%)	13 (18.3%)	0 (0.0%)	**0.035**
- Triple	25 (27.2%)	14 (19.7%)	11 (52.4%)	**0.003**
No data		8		
Aortic atherosclerosis No data	60 (64.5%)	47 (65.3%) 7	13 (61.9%)	0.776
Non-vascular CT-features				
Bowel luminal dilatation and/or air-fluid-levels No data	86 (92.5%)	66 (91.7%) 7	20 (95.2%)	0.585
Decreased or absent bowel wall enhancement No data	32 (34.8%)	25 (35.2%) 8	7 (33.3%)	0.874
Pneumatosis intestinalis No data	20 (21.5%)	18 (25.0%) 7	2 (9.5%)	0.225
Porto-mesenteric venous gas No data	9 (9.7%)	8 (11.1%) 7	1 (4.8%)	0.678

Data are presented as n (%), or median (IQR)

*SMA* superior mesenteric artery, *NASCET* North American Symptomatic Carotid Endarterectomy Trial

Bowel luminal dilatation and/or air-fluid levels were a common radiological finding in patients with AAOMI (total 92.5%, OR 91.7%, ER 95.2%; *p* = 0.585). In approximately one third of the patients, bowel wall enhancement was decreased or absent.

Pneumatosis intestinalis and porto-mesenteric venous gas (PMVG), considered as advanced signs of mesenteric ischemia, were more common in the OR group (25.0% and 11.1%) than in the ER group (9.5% and 4.8%), without significant differences (*p* = 0.225 and 0.678).

### Revascularization

Endovascular techniques for revascularization included 11 (52.4%) rotational thrombectomies, 13 (61.9%) aspiration thrombectomies, 15 (71.4%) PTAs, and 10 (47.6%) mesenteric stent placements. Lithotripsy was used in 2 cases (9.5%), and intra-arterial prostaglandin E1 was administered in 2 patients (9.5%).

In 4 out of 21 endovascular cases (19.0%), a single thrombectomy procedure (either rotational or aspiration) was performed; all of these were embolic occlusions. In another 4 (19.0%) embolic occlusions, adjunctive treatment with PTA and/or stent placement was required. The remaining 13 cases (62.0%), all of non-embolic origin, were treated using a combined approach involving balloon angioplasty, stent implantation and/or thrombectomy.

**Fig. 3:**
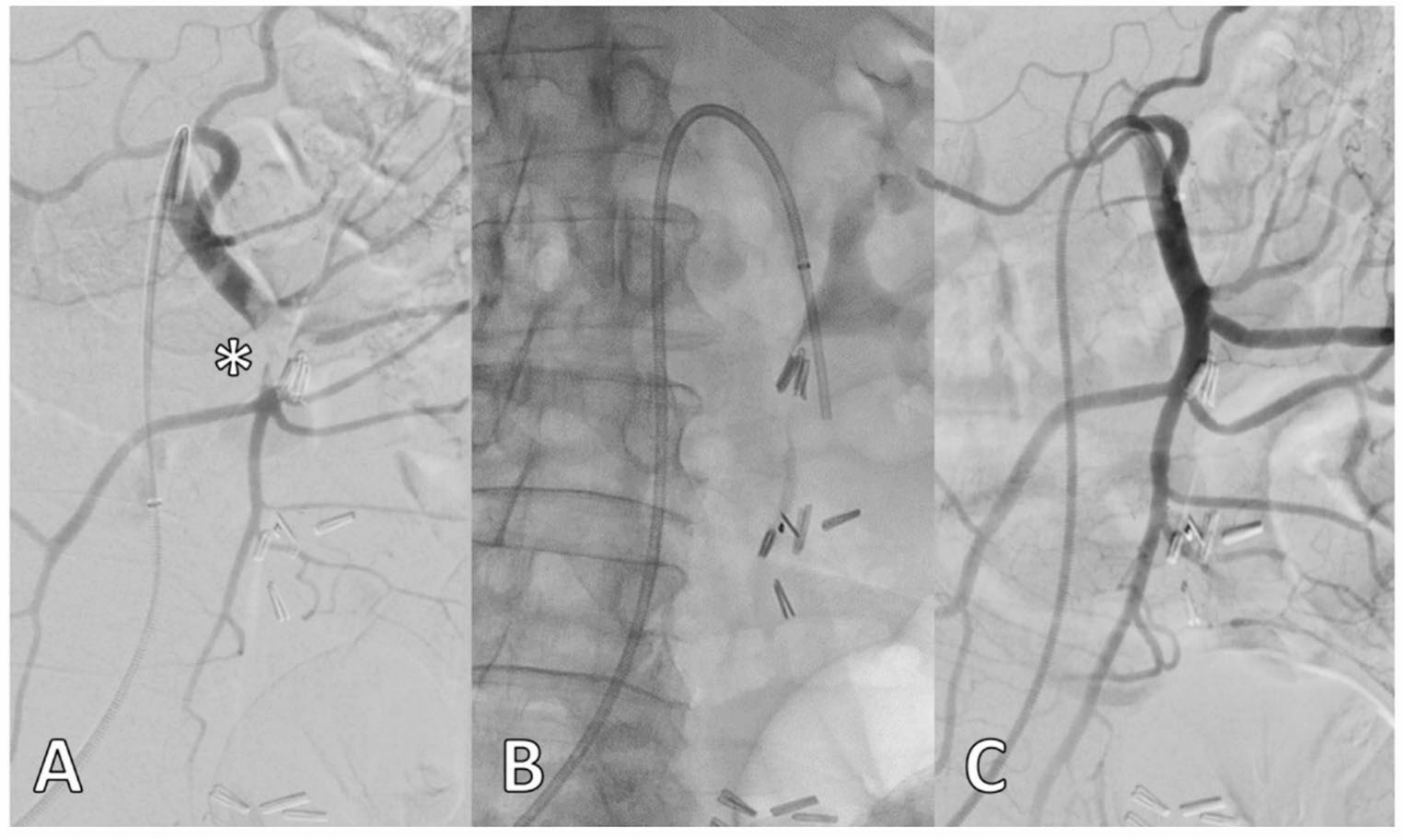
Endovascular revascularization procedure. Digital subtraction angiography demonstrates an embolic occlusion of the distal SMA involving jejunal and ileocolic arteries (asterisk) (**A**). Embolic material was removed using manual aspiration thrombectomy (**B**). The final angiogram shows successful recanalization of the SMA and restoration of mesenteric perfusion (**C**)

Figure 3 illustrates the sequence of an endovascular revascularization procedure.

Of the 21 endovascular cases, the target artery was the superior mesenteric artery in 17 patients (81.0%), both the SMA and the celiac artery (CA) in 3 patients (14.3%), and the CA alone in 1 patient (4.8%).

**Fig. 4:**
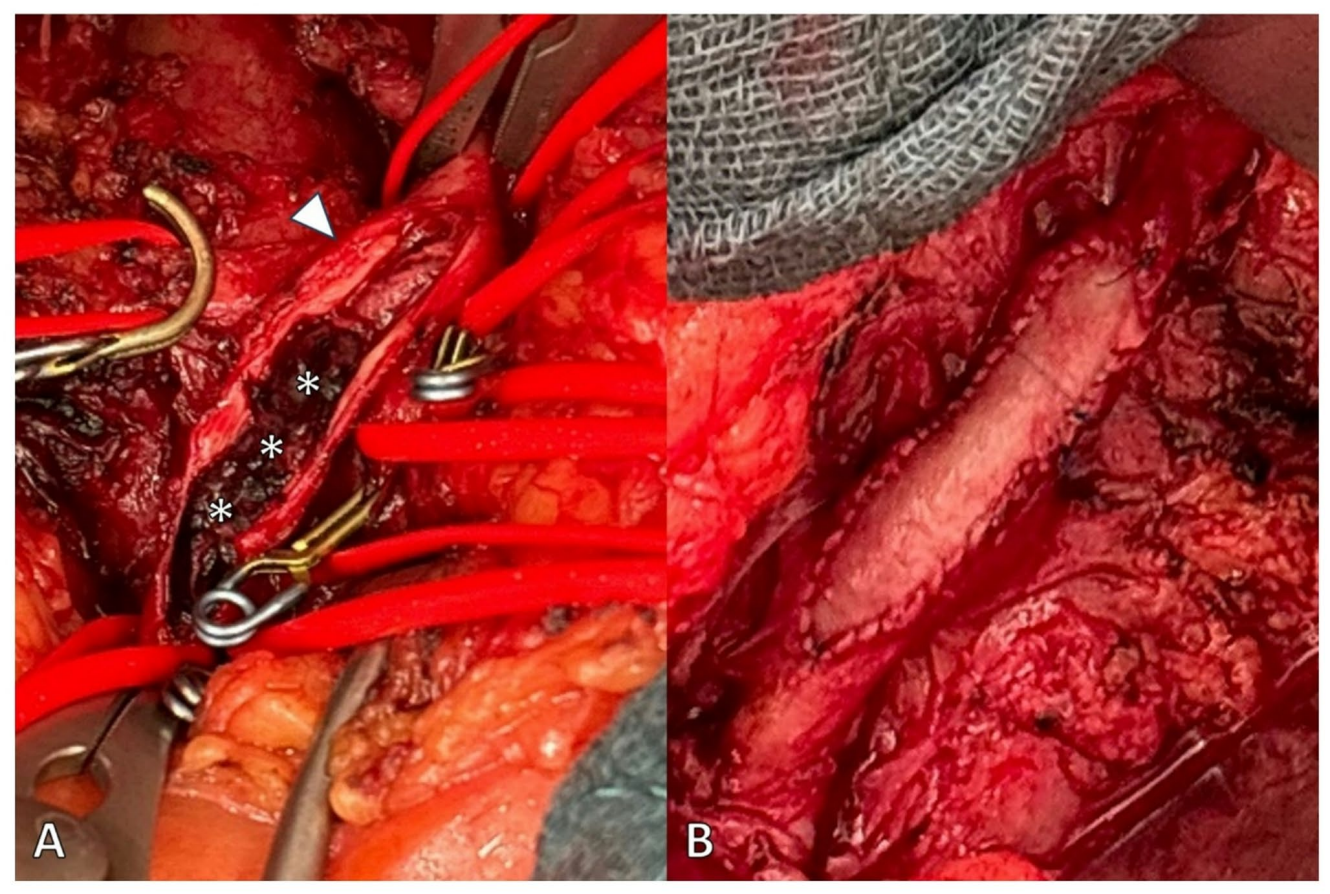
Intraoperative findings. Atherosclerotic stenosis with plaque rupture (arrowhead) and complete thrombotic occlusion (asterisks) are illustrated in (**A**). Treatment involved thrombendarterectomy followed by reconstruction using patch angioplasty with bovine pericardium (**B**)

Open vascular procedures performed in this cohort included 46 (58.2%) embolectomies, 4 (5.1%) TEAs, 27 (34.2%) mesenteric bypasses, and 2 (2.5%) ROMS. In Fig. 4, an intraoperative finding of an atherosclerotic stenosis with complete thrombotic occlusion is depicted, which was treated with TEA and patch angioplasty.

The technical success rate was 100% (79/79) in OR and 95.2% (20/21) in ER (*p* = 0.051). One patient with failed first-line endovascular therapy required open surgical revision and revascularization.

Primary vascular patency during hospitalization was 87.3% (69/79) in the open group and 90.5% (19/21) in the endovascular group (*p* = 0.694). One patient in both groups required an endovascular reintervention (*p* = 0.378). Vascular revision by open revascularization was necessary in two first-line OR patients and in one first-line ER patient (*p* = 0.511). Seven additional patients with initial open revascularization developed re-occlusion of the SMA, but no further vascular intervention was performed (*p* = 0.340). Among these seven patients, five had a non-salvageable abdominal situation with irreversible ischemia or septic deterioration and were transitioned to best supportive care. Two patients survived despite extensive intestinal resections, including one with long-term morbidity due to short bowel syndrome (Table 4).

**Table 4:** Revascularization

	Total *n* = 100 (100%)	Open *n* = 79 (79%)	Endovascular *n* = 21 (21%)	*p* value
Endovascular treatment				
Target artery				
Superior mesenteric artery			17 (81.0%)	
Celiac artery			1 (4.8%)	
Superior mesenteric artery + celiac artery			3 (14.3%)	
Inferior mesenteric artery			0 (0.0%)	
Endovascular technologies				
Rotational thrombectomy			11 (52.4%)	
Aspiration thrombectomy			13 (61.9%)	
Plain balloon angioplasty			15 (71.4%)	
Mesenteric stenting			10 (47.6%)	
Lithotripsy			2 (9.5%)	
Prostaglandin E1 for vasospasm			2 (9.5%)	
Selective thrombolysis with recombinant tissue plasminogen activator			0 (0.0%)	
Open revascularization				
Open vascular procedure				
Embolectomy		46 (58.2%)		
Thrombendarterectomy		4 (5.1%)		
Mesenteric bypass		27 (34.2%)		
Retrograde open mesenteric stenting		2 (2.5%)		
Technical success	99 (99.0%)	79 (100%)	20 (95.2%)	0.051
Vascular patency and reintervention				
Primary Patency	88 (88.0%)	69 (87.3%)	19 (90.5%)	0.694
Endovascular reintervention	2 (2.0%)	1 (1.3%)	1 (4.8%)	0.378
Open vascular revision	3 (3.0%)	2 (2.5%)	1 (4.8%)	0.511
Re-occlusion, no revision	7 (7.0%)	7 (8.9%)	0 (0.0%)	0.340

Data are presented as n (%)

### Surgical management

Exploratory laparotomy or laparoscopy was performed in all patients, with primary laparotomy being the predominant approach (total 93.0%, OR 98.7%, ER 71.4%). In six cases from the ER group, laparoscopy was the initial approach (4 patients received laparoscopy alone, while 2 cases required conversion from laparoscopy to laparotomy).

Overall, 34% of patients underwent bowel resection at primary abdominal exploration (OR 34.2%, ER 33.3%; *p* = 0.942). 42.9% (9/21) of ER patients required an ostomy, compared to 32.9% (26/79) of OR patients (*p* = 0.396). Damage control surgery was carried out in 24% of all patients (OR 25.3% vs. ER 19.0%; *p* = 0.775). A temporary abdominal closure system was used in 40% of cases (40.5% in open surgery vs. 38.1% in endovascular cohort; *p* = 0.841). Planned second-look laparotomy was performed in 53% of cases (OR 55.7% vs. ER 42.9%; *p* = 0.295), while unplanned relaparotomies occurred in 22.0% (OR 19.0% vs. ER 33.3%; *p* = 0.158). The median number of relaparotomies per group was 1 (range 0–5). Table 5 provides further information on the surgical management.

**Table 5:** Surgical management

	Total *n* = 100 (100%)	Open *n* = 79 (79%)	Endovascular *n* = 21 (21%)	*p* value
Abdominal exploration				
Laparotomy	93 (93.0%)	78 (98.7%)	15 (71.4%)	
Laparoscopy	4 (4.0%)	0 (0.0%)	4 (19.1%)	
Laparoscopy-to-laparotomy conversion	3 (3.0%)	1 (1.3%)	2 (9.5%)	
Bowel resection at primary abdominal exploration	34 (34.0%)	27 (34.2%)	7 (33.3%)	0.942
Type of bowel resection				
No bowel resection	48 (48.0%)	37 (46.8%)	11 (52.4%)	0.651
Small bowel resection < 100 cm	29 (29.0%)	22 (27.8%)	7 (33.3%)	0.623
Small bowel resection ≥ 100 cm	18 (18.0%)	15 (19.0%)	3 (14.3%)	0.757
Ileocecal resection	3 (3.0%)	3 (3.8%)	0 (0.0%)	1.000
Extended ileocecal resection	27 (27.0%)	20 (25.3%)	7 (33.3%)	0.462
Hemicolectomy left/right	16 (16.0%)	12 (15.2%)	4 (19.0%)	0.739
Extended colon resection	1 (1.0%)	1 (1.3%)	0 (0.0%)	1.000
Ostomy	35 (35.0%)	26 (32.9%)	9 (42.9%)	0.396
Intestinal anastomosis	21 (21.0%)	17 (21.5%)	4 (19.0%)	1.000
Damage control surgery	24 (24.0%)	20 (25.3%)	4 (19.0%)	0.775
Temporary abdominal closure system	40 (40.0%)	32 (40.5%)	8 (38.1%)	0.841
Second-look laparotomy, planned	53 (53.0%)	44 (55.7%)	9 (42.9%)	0.295
Re-laparotomy, unplanned	22 (22.0%)	15 (19.0%)	7 (33.3%)	0.158
Number of re-laparotomies, median (range)	1 (0–5)	1 (0–5)	1 (0–3)	0.562

Data are presented as n (%), or median (range)

### Mortality and outcomes

The in-hospital-mortality rate was 48.1% (38/79) of OR patients and 33.3% (7/21) of ER patients (*p* = 0.227).

42 patients (53.2%) with open surgical treatment required bowel resection, compared to 10 patients (47.6%) with endovascular first-line revascularization (*p* = 0.651). The median extent of bowel resection was 69 cm (IQR: 39–112) in the OR group and 71 cm (IQR: 62–116) in the ER group (*p* = 0.350).

Short bowel syndrome, defined by parenteral nutrition dependency at discharge, was observed in 12% of all patients, with no difference between the OR and ER groups (OR 12.7% vs. ER 9.5%; *p* = 1.000).

Median hospital stay was 15 days (IQR: 6–26) in the OR group compared to 11 days (IQR: 4–19) in the ER group (*p* = 0.484).

The median length of ICU stay was 5 days (IQR: 2–11) in the OR group and 4 days (IQR: 1–5) in the ER group (*p* = 0.172) (Table 6).

**Table 6:** Mortality and outcomes

	Total *n* = 100 (100%)	Open *n* = 79 (79%)	Endovascular *n* = 21 (21%)	*p* value
Morbidity				
Bowel resection	52 (52.0%)	42 (53.2%)	10 (47.6%)	0.651
- Colon resection	33 (33.0%)	25 (31.6%)	8 (38.1%)	0.576
- Small bowel resection	50 (50.0%)	40 (50.6%)	10 (47.6%)	0.806
- Ileocecal resection	30 (30.0%)	23 (29.1%)	7 (33.3%)	0.708
Extent of bowel resection (pathological specimen), median (IQR)				
- Overall, cm	69 (42–114)	69 (39–112)	71 (62–116)	0.350
- Colon, cm	15 (13–26)	15 (12–29)	16 (14–23)	0.786
- Small intestine, cm	55 (28–112)	66 (20–112)	54 (45–111)	0.687
Short bowel syndrome	12 (12.0%)	10 (12.7%)	2 (9.5%)	1.000
Tracheostomy	11 (11.0%)	8 (10.1%)	3 (14.3%)	0.698
Postoperative complications, CDC ≥ grade III	72 (72.0%)	56 (70.9%)	16 (76.2%)	0.630
Postoperative complications, CDC maximum grade				
Grade 0	17 (17.0%)	13 (16.5%)	4 (19.0%)	0.751
Grade II	11 (11.0%)	10 (12.7%)	1 (4.8%)	0.450
Grade IIIa	7 (7.0%)	3 (3.8%)	4 (19.0%)	**0.034**
Grade IIIb	7 (7.0%)	6 (7.6%)	1 (4.8%)	1.000
Grade IVa	11 (11.0%)	8 (10.1%)	3 (14.3%)	0.695
Grade IVb	2 (2.0%)	1 (1.3%)	1 (4.8%)	0.378
Grade V	45 (45.0%)	38 (48.1%)	7 (33.3%)	0.227
Length of hospital stay, days, median (IQR)	15 (6–24)	15 (6–26)	11 (4–19)	0.484
Length of intensive care unit stay, days, median (IQR)	5 (2–9)	5 (2–11)	4 (1–5)	0.172
Mortality				
In-Hospital Mortality	45 (45.0%)	38 (48.1%)	7 (33.3%)	0.227

Data are presented as n (%), or median (IQR)

*CDC* Clavien-Dindo classification

### Follow-up

The median follow-up (FU) period was 14 months overall (IQR: 2–30), with 13 months (IQR: 2–32) in the open revascularization group and 17 months (IQR: 3–29) in the endovascular revascularization group (Table 7). During FU, mesenteric re-occlusion or re-intervention occurred in 5.1% of OR patients and 19.0% of ER patients (*p* = 0.196). Death during FU was observed in 5.1% of OR patients and 9.5% of ER patients (*p* = 1.000).

**Table 7:** Follow-up

	Total *n* = 100 (100%)	Open *n* = 79 (79%)	Endovascular *n* = 21 (21%)	*p* value
FU, months, median (IQR)	14 (2–30)	13 (2–32)	17 (3–29)	
Mesenteric re-intervention/-occlusion in FU	8 (8%)	4 (5.1%)	4 (19.0%)	0.196
Death in FU	6 (6%)	4 (5.1%)	2 (9.5%)	1.000
12-month FU				
- Death	49 (49%)	41 (51.9%)	8 (38.1%)	0.442
- Survival	24 (24%)	17 (21.5%)	7 (33.3%)	
- LTFU	27 (27%)	21 (26.6%)	6 (28.6%)	

Data are presented as n (%), or median (IQR)

*FU *Follow-up, *LTFU* Lost-to-follow-up

At 12 months, mortality was 51.9% in the OR group and 38.1% in the ER group (*p* = 0.442). Survival at 12 months was 21.5% in the OR group and 33.3% in the ER group. The proportion of patients lost to follow-up was similar between the groups (26.6% in OR vs. 28.6% in ER).

## Discussion

Even if there have been some significant advances in diagnosis and therapy of acute mesenteric ischemia, it is still a life-threatening and complex vascular emergency encompassing with a high mortality rate of up to 70%. The poor prognosis is based on delayed diagnosis due to non-specific clinical symptoms and individual characteristics of patients suffering from AAOMI. Furthermore, there is still a lack of strong evidence for adequate therapeutical pathways in case of AAOMI.

In our monocentric retrospective study, 100 patients with AAOMI could be included. The study population had a median age of 75 years, frequent cardiovascular risk factors, and a median CCS of 5.5, indicating a severely comorbid and high-risk cohort. These characteristics align with previous reports showing that AAOMI predominantly affects elderly patients with pre-existing illnesses [4, 9]. The high mortality rate observed in our cohort (45%), despite the exclusion of patients who received primary palliation, those who had no revascularization, or those with NSBN at initial laparotomy, reflects the life-threatening nature of AAOMI.

Early diagnosis and urgent treatment are critical to improving outcomes in AAOMI [5, 10]. Achieving this requires increased clinical awareness to recognize the condition in its early stages, prior to the onset of transmural intestinal necrosis [11]. Several studies have demonstrated a survival benefit with prompt revascularization. For example, Chou et al. reported a 30-day mortality rate of 62% without revascularization compared to 42% in patients who underwent revascularization [12]. Similarly, Tran et al. showed a significantly lower 30-day mortality of 25% in patients receiving early revascularization versus 39.6% with delayed treatment [13]. More than half of our patients experienced delays exceeding 24 h, likely contributing to more advanced disease stages at the time of presentation and intervention.

The optimal first-line treatment, open surgical versus endovascular revascularization, remains a subject of ongoing discussion. A randomized control trial directly comparing the two approaches is currently lacking. Observational studies suggest benefits of endovascular therapy, including lower mortality and bowel resection rates [6–8]. Arthurs et al. reported mortality rates of 36% following successful endovascular treatment compared to 50% with traditional open surgery, with median bowel resections of 52 cm versus 160 cm, respectively [8]. A meta-analysis by El Farargy et al. confirmed improved 30-day mortality and reduced bowel resection following endovascular treatment [14].

Recent systematic reviews have evaluated outcomes of endovascular versus open surgical revascularization in AAOMI [15, 16]. One scoping review, encompassing 39 studies with 20,991 patients, reported lower 30-day mortality for endovascular interventions (0–53.8%) compared to open surgery (21–81%), including reductions in bowel resection and re-laparotomy rates, as well as shorter hospital and ICU stays [15]. A second systematic review including six retrospective cohort studies with 951 patients similarly identified lower in-hospital mortality, fewer bowel resections, preservation of intestinal length, and reduced rates of complications such as acute renal failure and pulmonary events following endovascular therapy [16]. However, both analyses were limited by pronounced selection bias, as patients with less advanced disease or without evidence of bowel necrosis at presentation were preferentially treated endovascularly, and by substantial heterogeneity among studies. These methodological limitations likely contributed to the observed outcome differences and underscore the need for cautious interpretation of the findings.

An important aspect highlighted by our data is the substantial impact of patient selection on treatment allocation. Nearly one third of patients treated endovascularly presented with in-stent thrombosis, which occurred significantly more often in the endovascular group than in the open surgery group (28.6% vs. 1.3%; *p* = 0.0003). This reflects the distinct baseline characteristics of the endovascular cohort and underscores that differences between treatment groups are strongly influenced by the underlying occlusion etiology.

Furthermore, the significantly higher proportion of open procedures performed outside regular working hours in our cohort (70.9% vs. 47.6%; *p* = 0.045) likely reflects the gradual implementation of endovascular therapy at our institution. As endovascular approaches evolved, adequate expertise, staff availability, and technical resources required time to develop. This observation provides real-world insight into how institutional logistics and experience influence treatment selection.

Overall, these findings contribute to the current evidence by illustrating (1) the real-world impact of occlusion characteristics on treatment selection, (2) the influence of institutional expertise and availability on procedural choice, and (3) the transitional nature of mesenteric revascularization over a 20-year period. These aspects are seldom addressed in previous retrospective studies and complement the conclusions of recent systematic reviews, highlighting the added value of our study in understanding practical determinants of treatment allocation.

In our cohort, patients undergoing endovascular revascularization showed numerical trends toward lower in-hospital mortality (33.3% vs. 48.1%), fewer bowel resections (47.6% vs. 53.2%), and shorter hospital and ICU stays (median 11 days vs. 15 days and 4 days vs. 5 days, respectively). None of these differences reached statistical significance, likely due to limited sample size and inherent selection bias. Patients with advanced disease markers, such as peritoneal irritation, pneumatosis intestinalis, or complex occlusions, were more frequently treated with open surgery. This pattern reflects the substantial influence of clinical severity and vascular morphology on therapeutic decision-making [17]. The trends observed in our cohort are consistent with those reported in previous studies; however, they did not reach statistical significance, and no conclusions regarding treatment superiority can be drawn.

Our protocol of combining endovascular intervention with immediate laparotomy or laparoscopy allows timely assessment and management of ischemic bowel, addressing concerns regarding potential delays in bowel viability evaluation after endovascular therapy [4].

As a retrospective single-center study with an imbalance in group sizes (21 endovascular vs. 79 open surgical patients), the generalizability and statistical power of comparisons are limited. Selection bias remains a potential confounder since patients with more advanced ischemia were more likely to undergo open surgery. Furthermore, no separate analysis was performed for embolic versus non-embolic etiologies. This distinction may be clinically relevant, as thrombotic or atherosclerotic mesenteric ischemia is often associated with chronic hypoperfusion and well-developed collateral circulation, potentially allowing for greater ischemia tolerance and a longer therapeutic window compared to embolic occlusion [17].

The establishment of dedicated mesenteric stroke centers with standardized, multidisciplinary protocols could improve patient outcomes. Early recognition and intervention are paramount to reduce mortality [5, 10]. Endovascular and open surgical approaches should be considered complementary, with individualized treatment decisions based on patient condition, anatomic factors, and institutional expertise [18]. Future research should focus on stratifying AAOMI subtypes (embolic, thrombotic, in-stent thrombosis, and atherosclerotic), evaluating specific treatment techniques, and incorporating clinical status to optimize management.

## Conclusion

Given the limitations of the relatively small sample size, the influence of patient selection, differences in occlusion type and ischemic severity, as well as the impact of institutional expertise, open surgical and endovascular revascularization achieved comparable outcomes in our retrospective cohort. Treatment decisions should therefore be individualized, considering the underlying occlusion etiology, the patient’s clinical condition, and the available institutional expertise. Establishing an interdisciplinary institutional protocol is essential to optimize the management of this time-critical condition. Further prospective multicenter studies are warranted to better delineate optimal treatment strategies for mesenteric revascularization.

## Data Availability

No datasets were generated or analysed during the current study.

## Abbreviations

AAOMI: Acute arterial occlusive mesenteric ischemia
CA: Celiac artery
CCS: Charlson comorbidity score
CRP: C-reactive protein
CT: Computed tomography
DSA: Digital subtraction angiography
ER: Endovascular revascularization
FU: Follow-up
ICU: Intensive care unit
IQR: Interquartile range
NSBN: Non survivable bowel necrosis
OR: Open surgical revascularization
PTA: Percutaneous transluminal angioplasty
PMVG: Porto-mesenteric venous gas
ROMS: Retrograde open mesenteric stenting
SMA: Superior mesenteric artery
TEA: Thrombendarterectomy
